# Scoping review and thematic analysis of informed consent in humanitarian emergencies

**DOI:** 10.1186/s12910-024-01125-w

**Published:** 2024-11-20

**Authors:** Benjamin Thomson, S. Mehta, C. Robinson

**Affiliations:** Bloomberg School of Public Health, Johns Hopkins University, Baltimore, USA

**Keywords:** Humanitarian emergency, Informed consent, Humanitarian research, Research ethics

## Abstract

**Background:**

To identify and to summarize challenges related to the informed consent process for research completed during humanitarian emergencies.

**Methods:**

Using relevant search terms, a search of 5 databases was completed, without language, date, or study type restriction. Studies were screened for inclusion, with eligible studies being those that were relevant to the informed consent process for research studies completed in humanitarian emergencies. A Grounded Theory Analysis was completed to identify themes and subthemes.

**Results:**

Review identified 30 relevant studies. We identified 11 challenges (lack of trust, therapeutic misconception, reduced capacity, security and privacy concerns, harmful research, power differential, literacy, language/local and cultural context, researcher burden and re-evaluation of ongoing trials) and 7 strategies (engage local research communities, use alternative to standard written consent process, modify traditional process of research ethics board review, dynamic consent, training of research staff, mandating transparency of commercial interests, and mandating reporting of informed consent process in all publications) to confront the challenges. These challenges and strategies were unique to the informed consent process in research conducted during humanitarian emergencies.

**Conclusions:**

This scoping review identified an evidence-based guide for researchers and research ethics boards to perform ethical informed consent procedures in humanitarian emergencies.

**Trial Registration:**

This trial was not registered as scoping reviews can not be registered as per updated PROSPERO guidelines.

## Background

The rate of climate and weather-related disasters has increased almost 35% since 1990 [1]. Research on humanitarian disasters is therefore critically needed to enhance delivery of services and resources, to assess vulnerability of individuals and communities, to improve acute and chronic disease management, and to enhance preparedness, response and recovery efforts [2]. Despite the need, there continues to be a large evidence gap on how organizations should respond to humanitarian emergencies [3, 4].

A humanitarian emergency is defined as an event or series of events that represents a critical threat to the health, safety, security or wellbeing of a community or other large group of people, usually over a wide area.

United Nations Human Rights Office of the High Commissioner [5].

Significant efforts have grown to confront this evidence gap, including the collaboration between the London School of Hygiene and Tropical Medicine, and the Johns Hopkins Center for Humanitarian Health, to regularly assess evidence for humanitarian health interventions in Low- and Middle-income countries (LMIC) [6]. Research performed during humanitarian emergencies raises unique ethical considerations, including the vulnerability of the victims of the humanitarian response [7], and pressure from national and international government and non-government stakeholders. There is significant time-sensitivity of interventions, and thus often a need to expedite institutional review board (IRB) approval [8]. Finally, the research needs to be completed in a culturally appropriate way, despite often having limited history with the community [9]. 

There have been efforts to establish research ethics standards in humanitarian emergencies. The Council for International Organizations of Medical Sciences (CIOMS) includes refugees or displaced people in the consideration of vulnerable groups. However, there is nothing more specific regarding additional vulnerable population groups in humanitarian emergencies, such as political dissidents, undocumented persons or unaccompanied minors [10]. Similarly, ELHRA has developed a ethics framework for research in humanitarian crises [11]. Unfortunately, the section on informed consent is limited, and this report is not updated since 2017. As such, a focused evaluation of informed consent in research in humanitarian emergencies is needed.

The Belmont report outlined three essential principles for research involving human subjects, being respect for persons, beneficence and justice [12]. Application of these principles mandates informed consent of study participants, which *“can be analyzed as containing three elements: information*,* comprehension and voluntariness.*” Therefore, fully respecting these three principles can be challenging in a humanitarian crisis, where there are unique conditions such as time-sensitivity of interventions and vulnerability of research participants. The aim of this study was to identify and to summarize challenges related to the process of informed consent in humanitarian emergencies.

## Methods

This review was guided by the methodological framework proposed by the Joanna Briggs Institute [13]. As per this framework, the research question was what is known regarding the process of informed consent in research performed in humanitarian emergencies. The identification and selection of relevant studies, extraction and summary of the data, and reporting of the results are described below.

### Search Strategies and Study Identification

Online databases were searched from inception to November 20 (2023), and included MEDLINE, Embase, Cochrane Central Register of Controlled Trials, Web Of Science and Scopus. The search strategy was not limited by language, or year. Search terms included “humanitarian crisis,” “humanitarian emergency,” “humanitarian event”, “humanitarian disaster”, “public health emergency of international concern”, “research consent”, “informed consent”, and “research ethics.” Eligible studies included those that were peer-reviewed publications, which identified a challenge related to the informed consent process for human subjects, for research in humanitarian emergencies. We defined humanitarian emergency as per the United Nations Human Rights Office of the High Commissioner: [5]

### Study Selection

Search results were imported into Covidence systematic review software (Veritas Health Innovation, Melbourne, Australia). Two reviewers (SM and BT) independently screened titles and abstracts, and independently reviewed manuscripts for inclusion. Disagreements between reviewers at each stage were resolved by discussion and consensus.

### Data Extraction and Synthesis

A data extraction form was developed a priori. Study characteristics included the type of study, location, type of humanitarian emergency, intervention and outcomes. Study location for literature or systematic review articles was determined by the primary author country affiliation. Other study types’ (interview, survey, etc.) location was determined by the country in which the research was conducted. All types of interventions and outcomes were considered.

Notes were made about each included study, and are available on request to the corresponding author of this manuscript.

### Thematic analysis and Code Generation

Study type, intervention, and outcomes were too heterogenous to be combined across studies.

Study interventions and outcomes were assessed using constant comparative method, as previously described [14]. Study topics identified in notes were evaluated and codes generated. These codes were further refined and searched for themes, which were then defined and named. A list of themes and subthemes was finalized.

### Ethics

Ethics approval was not required for this study, since all data was collected anonymously or from previously published sources.

## Results

### Selection of Sources of Evidence

**Fig. 1:**
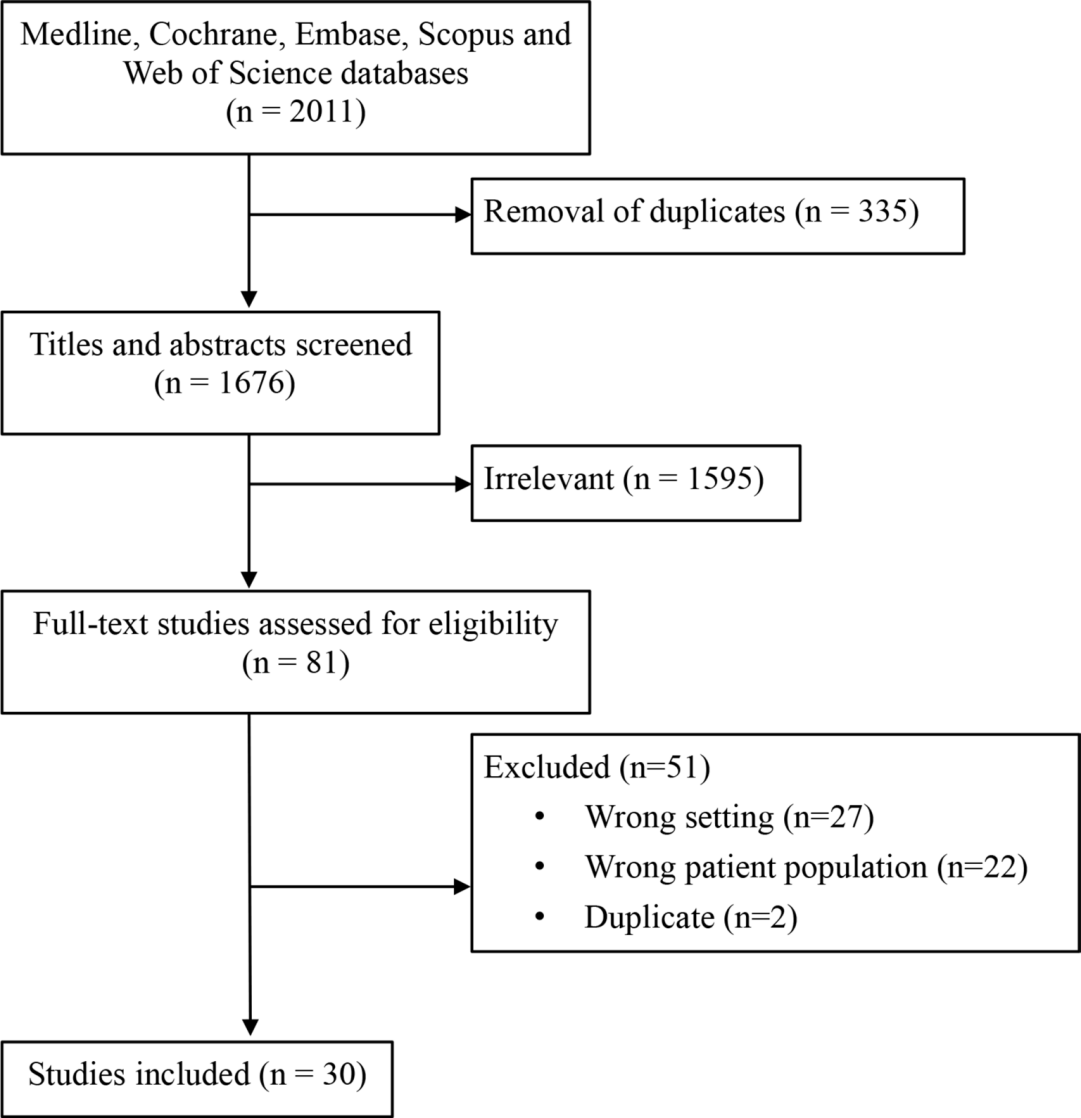
Prisma flow diagram for review

Database search yielded 2011 references (Fig. 1). Removal of duplicates led to 1676 total references. Of these, 1595 were deemed to be irrelevant to the aim of the review. There were 81 articles for full-text review. After full text review, 30 studies were included (Table 1).

**Table 1:** Study Characteristics

Study	Type of study	Study Location	Humanitarian Context/Focus	Intervention
Aktar, [55]	Review of research study	Bangladesh	Refugee (Bangladesh) camps of Rohingya refugees	None
Allden, [16]	Literature review	USA	Mental health research in emergency settings (international)	None
Ataullahjan A et al., [91]	Systematic review	Canada	Why is stricter oversight needed in research in conflict settings (international)	None
Browne JL et al., [64]	Systematic review	Netherlands	Willingness to participate in research in LMIC (international)	None
Bruno W et al., [35]	Systematic review	USA	Ethics of conducting research in humanitarian settings (international)	None
Chiumento A et al., [2]	Literature review	UK	Mental health research in humanitarian settings (international)	None
Eckenwiler, 2015	Fictional case review	USA	Conflict-affected areas	None
Eid-Heberle K, [71]	Literature review	USA	Nursing research in post-disaster phase (international)	None
Falb K et al., [46]	Literature review	USA	Recommendations for research ethics boards in crisis settings (international)	None
Glass N et al., [90]	Program Evaluation	USA	Program targeting Gender-Based violence in Conflict and Displacement (international)	None
Gobat NH et al., [82]	Systematic review	UK	Epidemic/Pandemic research preparedness (international)	None
Hunt M et al., [8]	Interview	Canada	Natural disasters in LMICs (international)	Interviews of Ethics Board Members
Hunt M et al., [30]	Systematic review	Canada	Ethical issues of providing mental health in disasters (international)	None
Hussein G et al., [86]	Systematic review	UK	Armed conflict in Darfur (Sudan) (2004–2012)	None
Jegede AS, [27]	Review of research study	Nigeria	Meningitis outbreak in Nigeria, Pfizer Trovan trial	None
Kurihara C et al., [60]	Editorial	Japan	Pre-existing trials (Ukraine) disrupted by humanitarian crisis	None
Lavin RP et al., 2012	Literature review	USA	Research during Disasters (international)	None
Maglio F et al., [41]	Literature review	UK	Children’s participation in research in humanitarian settings (international)	None
McGowan CR et al., [15]	Survey	UK	Ebola Outbreak (West Africa)	Survey of Responders to consent process
Mena, [51]	Review of research study	Netherlands	Conflict-affected areas (South Sudan and Afghanistan)	None
Mfutso-Bengo J et al., [37]	Literature review	Malawi	Humanitarian emergencies (international)	None
O’Mathuna D, [62]	Literature review	Ireland	Humanitarian emergencies (international)	None
Parkinson, [21]	Literature review	USA	Conflict-affected areas (international)	None
Perakslis ED, [53]	Literature review	USA	Humanitarian settings (international)	None
Pincock, [51]	Review of research study	UK	Refugee research settings (Uganda)	None
Pringle, [29]	Literature review	Canada	Research in humanitarian settings (international)	None
Roth DE, [59]	Editorial	Canada	Research in humanitarian settings (international)	None
Schopper D et al., [45]	Report of Ethics Board Logistics	Switzerland	MSF Research Ethics Board	None
Tansey, [20]	Interview	Canada	Research in humanitarian settings (international)	None
Yimer G et al., [22]	Review of research study	USA	Internal displacement in Ethiopia (CAGED study)	None
Abbreviations: LMIC = Low and Middle Income Countries; MSF = Medecins Sans Frontieres; NS/NR = Not stated or not reported				

### Study Characteristics

The 30 studies included literature reviews (*n* = 11), systematic reviews (*n* = 6), reviews of research studies (*n* = 5), editorials (*n* = 2), interviews (*n* = 2), fictional case review (*n* = 1), program evaluation (*n* = 1), survey (*n* = 1) and review of Ethics Board logistics (*n* = 1) (Table 1). All studies were relevant to research in humanitarian settings. Only 2 studies included an intervention, one of which was a survey of people who had previously consented to an online consent process prior to responding to an Ebola outbreak [15], and the other was an interview of members of a research ethics board in Canada regarding consent processes for research performed during natural disasters [8]. 

Studies were completed in USA (*n* = 10), Canada (*n* = 6), UK (*n* = 6), Netherlands (*n* = 2) and one each in Bangladesh, Nigeria, Japan, Malawi, Ireland and Switzerland. The humanitarian context was international in most (*n* = 22) studies but focused on specific geographic region in other (*n* = 7) studies. One study used a fictional case review and thus did not reflect an actual geographic area.

### Thematic analysis and Code generation

The thematic analysis performed as part of this study led to two potential formats. The first format broke down informed consent procedures by the type of humanitarian emergency (e.g. Natural disaster, conflict, etc.). The second format organized by challenges and solutions, which provided a more logical and practical framework that could be applied to all types of humanitarian emergencies. Given the more universal applicability of the second format, it was chosen for the final themes.

Evaluation of notes for each study yielded two major themes, being the challenges (coded C) associated with informed consent in humanitarian emergencies, and the strategies (coded S) to overcome these challenges. Each theme was further studied to establish similarities, and a list of Challenges (C) (Table 2) and Strategies (S) themes (Table 3) were finalized.

**Table 2:** Challenges of informed consent in research in humanitarian emergencies

Challenge	Explanation	Studies (*n*)
C1: Lack of trust	There is a lack of trust between the potential research participants and the research team.	10
C2: Therapeutic misconception	Potential research participants believe that receiving humanitarian aid may require participation in research trial.	7
C3: Vulnerable population	Potential research participants are more vulnerable to coercion.	14
C4: Reduced capacity	Potential research participants have reduced capacity to be informed during the consent process.	3
C5: Security and Privacy concerns	Potential research participants need to maintain anonymity, and sharing identifying information may risk self or family.	12
C6: Harmful research	The research topic itself may retraumatize the potential research participants or be harmful to the community.	6
C7: Power differential	The research staff may be seen as an authority mandating research participation.	8
C8: Literacy	Potential research participants may not be literate.	6
C9: Language, local and cultural context	The informed consent process may not be tailored to the local language or dialect, or local and cultural contexts.	14
C10: Researcher burden	Researchers in humanitarian settings have limited timelines to initiate a research study, and traditional Research Ethics Boards may cause delays.	1
C11: Re-evaluation of ongoing trials	Research trials that are ongoing at the start of a humanitarian emergency require re-evaluation of the informed consent process. Also, potential research participants’ situation changes throughout humanitarian emergencies, and thus informed consent may need to be repeated.	6

**Table 3:** Strategies to Overcome Challenges of Informed Consent in Humanitarian Emergencies

Solution	Explanation	Studies (*n*)
S1: Engage local research communities	Involve the local community in research planning, recruitment, and operations.	14
S2: Use alternative to standard written consent process	Consider mechanisms of consent that are not written, or atypical in other ways	14
S3: Modify traditional process of Research Ethics Board Review	Modify the process of evaluating research performed in humanitarian emergencies, separately and differently from other trials	7
S4: Dynamic consent	Informed consent should be deliberately re-evaluated during an ongoing trial, and mechanisms built in trial design to facilitate it.	7
S5: Training of research staff in consent ethics	Staff completing the informed consent procedure should be trained in research ethics and the challenges in informed consent	7
S6: Mandate transparency of commercial interests when present	When a research study’s objective is to validate use of a drug or medical device for eventual profit motive, this should be explicitly stated	1
S7: Mandate reporting of informed consent process in all publications	All published studies should report the informed consent process.	2

### Themes- Challenges and Solutions

The most frequently identified challenge identified was tailoring the informed consent process to the local language and cultural context (C9, *n* = 14). This was seen in a variety of settings, including both internally displaced and refugee populations, natural disasters and epidemics and conflict settings, with children and adults, and in both response and recovery phases of emergency response. Potential solutions might include engaging local research communities to modify informed consent processes to be more appropriate for the local context (S1, *n* = 14), or using an alternative to standard written consent processes (S2, *n* = 14). Modifying written consent processes may also be helpful when literacy of research participants is an issue (C8, *n* = 6).

Vulnerable potential research participants being more vulnerable to coercion was also a frequently identified challenge (C3, *n* = 14). Vulnerable patient groups identified included children (orphaned or unattached), refugees or internally displaced persons, undocumented migrants, political dissidents, ethnic or religious minorities, victims of violence or human rights abuses, survivors of human trafficking, pregnant women, people with physical or mental disabilities.

The desire to maintain anonymity to assure safety, and concerns that information sharing may risk safety of self or family was commonly identified (C5, *n* = 12). This was seen in the context of a research participant’s concern regarding the possible publicization of his or her mental health status, undocumented status, political or religious views, or prior participation in conflict. This was also considered when ongoing participation in a research trial may delay safe evacuation. In this scenario, ongoing research participation may also put the safety of local research staff at risk. The release of demographic information that confirms a low proportion of military-age men was perceived in one study to put women, children and elderly persons in that geographic area at risk of violence from armed factions.

Lack of trust between potential research participants and the research team was frequently identified (C1, *n* = 10). Trust was identified as a reason for participation, and distrust as a reason for non-participation in research. Regions that have experienced historical ethical violations in one research program, may have generalized distrust against future research. Research that had revealed identities of women who were sexually assaulted, or the identity of activists or refugees, were identified as harmful research which led to longstanding community distrust. Similarly, an unethical trial in Northern Nigeria (Pfizer Trovan) led to generalized community distrust in health authorities, and by association, researchers. In each case, engagement of the local communities (S1) was recommended as a potential solution.

Given the limited timelines to initiate a research study in a humanitarian setting, the researcher burden to secure research ethics board (REB) approval, then research participants’ informed consent, was identified as a significant challenge (C10, *n* = 1). One remedy would be to modify the traditional process of REB approval to facilitate accelerated evaluation and approval or research protocols (S3, *n* = 7).

Given humanitarian emergencies are situations in which the safety of research participants may change, the risks of continuing in a research project also change (C11, *n* = 2). This mandates the need to re-evaluate informed consent throughout the research using dynamic informed consent (S4, *n* = 7).

## Discussion

### Challenges C1: Lack of Trust

Lack of trust in the research team is a key element in potential research participants choosing not to participate in research studies [16–21]. This is exacerbated in humanitarian emergencies, where regime change can lead to a generalized decrease in community trust of non-community members [22]. Potential research participants may become hesitant to sign forms, assuming that information is being collected for nefarious purposes [23], or that a signature signifies legal accountability [24]. 

In Ethiopia, the Campylobacter Genomics and Enteric Dysfunction (CAGED) research project provided an excellent example regarding the impact of the lack of trust. The CAGED study evaluated pathways of Campylobacter infections in children, which associate with adverse child health outcomes. The CAGED project initial study confirmed the prevalence of stunting in Campylobacter colonized children in rural Eastern Ethiopia to be 41% [25]. A longitudinal study was planned to enhance understanding of Campylobacter reservoirs and transmission pathways [26], but this study was disrupted by regional ethnic tensions and violence. Some regions became quite suspicious of outsiders, and several researchers became victims of violence. Many research participants withdrew their consent, necessitating the CAGED research team to plan community trust-building strategies [22]. 

The impact of not considering community trust prior to engaging in research in humanitarian settings can be profound. In 1996, Pfizer pharmaceuticals tested a new antibiotic Trovafloxacin (Trovan) during a meningitis epidemic in Nigeria. Key information was withheld in the informed consent process, and patients in the control arm were given a significantly lower dose of the standard treatment drug [27]. This led to anger and mistrust in future research trials, and was a significant contributing factor to the boycott of polio vaccination programs in 3 Northern Nigerian states in 2003 [28]. 

Distrustful study participants may reluctantly participate, leading to superficial engagement, providing either incomplete or dishonest responses, rushing through survey questions, and providing limited information [21]. 

### C2: Therapeutic misconception

Potential research participants may be unclear about the distinction between research and humanitarian aid [16, 21, 29, 30]. In humanitarian emergencies, there is a heavy reliance on humanitarian actors to provide food, protection, education, housing, social and medical services; this may unintentionally coerce people to participate in research [21, 31]. This phenomena has been referred to as the “therapeutic misconception,” [32] or the “dual imperative” of research and benefiting local communities [22]. The lack of comprehension usually works in the favor of researchers, since potential participants may want to receive a treatment or humanitarian aid, and think they must participate in the research study to do so [27]. 

### C3: Vulnerable population

People who are victims of a humanitarian emergency experience disrupted community and social networks, threats to basic necessities such as food and shelter, a variety of serious public health risks, and human rights abuses including gender-based violence [16, 20, 33, 34]. Weak government systems and insecure living conditions are particularly high risk conditions for inadequate research consent processes and coercion [35]. People who are fleeing governments or non-state actors involved in coercive practices may not recognize they can decline participation in research studies [29, 36]. Research participants should be considered independent moral agents that are able to exercise autonomy, but given these vulnerabilities, additional measures need to be instituted to assure protection [37]. 

Children in humanitarian emergencies present a unique ethical challenge. They often do not have the legal right to consent, and they may be incapable of understanding some or all of the research content necessary for informed consent [38]. Where children are considered legally unable to provide informed consent, a substitute may be to document the child’s assent, simply the agreement to participate [39]. However, when a parent or legal guardian is available, their informed consent should supplement the child’s assent. Also, provision of assent from a child does not negate researchers’ obligation to inform that child of the risks of participation in research [40]. 

During a humanitarian emergency, children may be separated from their parents or caregivers, and researchers attempting to attain informed consent may be untrained in the detection of childhood distress [41]. Unaccompanied minors may sometimes be able to consent for themselves, although special protection may be required by involving national authorities, and additional psychological and legal supports may be needed [42]. On the other hand, excluding vulnerable children from research in humanitarian settings may withhold the opportunity to benefit from the research, and might limit research that could enhance their outcomes in such settings [41]. 

An academic collaboration between New Zealand Universities, Childwatch International Research Network and Unicef, called the Ethical Research Involving Children compendium, provides some guidance [40]. Even in humanitarian emergencies there are particular groups that necessitate more consideration given additional vulnerabilities. In addition to children, these include refugees, internally displaced persons, undocumented migrants, ethnic or religious minorities, political dissidents, survivors of human rights abuses, people with disabilities, and survivors of human trafficking [43]. 

### C4: Reduced capacity

In ideal circumstances, potential research participants should be fully appraised and aware of the research conditions [44]. This requires time to discuss and to consider the information with friends and family, but these social networks are often disrupted in crisis [30]. Victims of humanitarian emergencies may be traumatized, and thus have reduced capacity to process information required for informed consent [30]. This may be further exacerbated with victims of sexual violence, torture, or people with disabilities [45]. 

### C5: Security and Privacy Concerns

Humanitarian emergencies commonly have ethnic or political tensions, so sharing of sensitive information can be seen as a risk. Data collection poses perceived or actual risks of confidentiality, especially when questions include identification of gender, religion, migration status, social or political affiliation [16, 20, 46]. A breach of confidentiality by researchers could bring significant security risks to research participants [47]. Written consent documents reveal the identity of research participants, which may indicate their membership of a particular social, political or military group [46]. Research participants may be the victims, perpetrators or witnesses of illegal activities and are thus at risk of losing anonymity and security, when signing a written consent form [48]. 

Recording the voice or photographing potential research participants can also accentuate concerns of anonymity and privacy concerns, and in younger people can raise concerns regarding child protection [41]. 

Sometimes, more data might be collected than researchers or participants recognize. For example, studies that use electronic capture of data may passively record the GPS coordinates of the informed consent location; this data could potentially be used by other parties if the electronic devices are not adequately protected [49]. 

### C6: Harmful research

Participation in a research trial may put a community or community leaders at risk, if there isn’t approval by relevant government or military authorities, or if the trial participants are asked to comment on resources provided by those authorities [34, 35]. 

Some research studies may precipitate trauma in participants, such as those on sexual violence, torture, sexual orientation, gender minorities, or people with different migration statuses. These studies are often in communities without resources to support those who are interviewed [16, 45]. A consequentialist approach can evaluate research harms and benefits, and assess how harms to research participants might be managed once the research is completed [40, 50]. Where research participants have nowhere to be referred after the research is completed, if the research causes harm, participation in the research trial may be unethical.

### C7: Power differential

A research team member is in a powerful position over potential research participants, in terms of safety, knowledge, economic stability, dependence on aid, and vulnerabilities [16, 51]. This power differential may lead to victims of humanitarian emergencies becoming victims of harmful clinical trials [27]. Uneven power dynamics are an obstacle to meaningful informed consent as the potential research participant may feel unable to ask the research team member questions to clarify the parts of the proposed research area to which he or she feels discomfort or uncertainty [46, 52]. 

### C8: Literacy

Potential research participants may have varying levels of literacy, thus rendering the written consent procedure ineffective [46]. Illiteracy makes communication of the research protocols and risks challenging, especially when the research protocols are complex [53]. Modifications in the informed consent procedure are required [2]. 

### C9: Language, local and cultural context

Comprehension of the informed consent procedure is influenced by several factors that include language, and cultural differences between researchers and potential participants [41, 53]. Research performed within multiple communities in close proximity may need to modify the informed consent process to overcome differences in local dialects [54, 55]. These challenges are increased with more complex research protocols [53]. 

A common challenge in research performed in low- and middle income countries is the difference between researcher and potential research participants’ understanding of illness. While European and North American researchers usually perceive illness as a more individualized problem, some cultures deem illness to be based more in community, spiritual or natural perspectives [56]. 

Written consent may be culturally, politically, historically or legally inappropriate in some societies in which collective decision making is done as a community rather than individually [2]. In these communities, community leaders may act as gatekeepers to support community members’ individual participation in a study. A lack of support from community leaders may place research staff at risk of subsequent harm [57]. 

### C10: Researcher burden

The informed consent process in humanitarian contexts poses unique challenges to the researcher which shouldn’t be overlooked. Researchers have limited time to prepare for a research trial in the acuity of a humanitarian emergency [34]. Researchers cannot proceed to consent potential research participants until the IRB has approved of the research protocols, but traditional IRBs often take a lot of time, with meetings too irregular to accommodate the timelines required to set up a research trial urgently in a humanitarian setting [58]. As the demands on researchers through IRBs increases, the likelihood that research will take place in humanitarian settings decreases [59]. 

### C11: Reevaluation of ongoing trials

Humanitarian emergencies should not be considered as a single moment in time, but instead as an evolution of changing economic, social, political and health factors, that constantly modifies a person’s risk of ongoing participation in a research trial. The disruptiveness and urgency of humanitarian crises force investigators to consider research participants’ health situation and vulnerabilities, as well as the extent to which ongoing participation is the optimal way to address health needs [60]. 

The recent war in Ukraine caused dramatic disruption of several ongoing clinical trials, and modified the safety and vulnerabilities of research participants [60]. Recognizing the need to continue ongoing support of clinical trial participants, to maintain supplies of medications, and to address safety issues, the Ukrainian Clinical Research Support Initiative (UCRSI) was created urgently. The consent process was reconsidered since information and patient context, with ongoing research participation risks, had changed. Similar changes in risks of ongoing participation in research trials, in humanitarian emergencies, should similarly precipitate reevaluation of the informed consent procedure. For example, a change of governmental or non-governmental authority may modify the risk of being identified, or of being less mobile to participate in the trial [35]. 

### Discussion: *Strategies S1: Engage local research communities*

The 2015 CIOMS International Ethical Guidelines for Health-Related Research recommends community engagement when research is done in a low-resource setting [61]. This is a well-founded recommendation for at least five reasons. Firstly, having local community members on the research team may improve research infrastructure, capacity [2], and trust [16, 29, 34, 62]. Secondly, complex research trials often require community engagement to develop sufficient understanding to be informed in the consent process [2, 20, 55]. The consent process must be adapted to assure context for potential research participants and this may be particularly challenging in refugee camps with multiple cultural, linguistic, national and religious backgrounds [46]. This adaptation can be facilitated by local translators, or people who understand the local culture and community context [62]. Thirdly, when there is a potential for community harm in participation of the research project, community consent may be necessary prior to the recruitment of individual research participants [45]. For example, when a research trial evaluates a governmental response to a humanitarian emergency by interviewing community members, that community may later be targeted by government action. Alternatively, some program interventions occur at the community level, so consent of every individual in that community can be replaced with consent from community leaders or elders [37]. Fourthly, engaging communities decreases the impact of vulnerability and socioeconomic factors on the consent process, by addressing the expectation of personal and community benefits, and enhancing the understanding of the research [63, 64]. Fifthly, increasing community participation narrows the power differential and knowledge gap between research team members and research participants [52, 65]. 

As described in Yimer et al., changes in governmental powers during a study in Ethiopia led to distrust of the research staff in the middle of the study, with several research participants withdrawing consent, in addition to research staff being attacked. In recognition that trust needed to be built, the research team engaged communities by meeting village leadership, and spending time in prayer activities. Community elders communicated positively about the research and research team, noting that the research team had similar language and religion. It was reinforced that the research team were independent of government officials and instead worked at the university. The research team offered support to reach medical assistance in addition to continuing the research survey. Furthermore, the research team created community advisory boards that were critical to modifying the survey to resolve conflicts of dialectical differences in meaning of the survey [22]. 

It can also be helpful to have 2 institutional review boards, one at the researcher site and the other from a local nongovernmental organization. This enhances community trust, and enhances the local context of the research study [59, 66]. 

In a humanitarian emergency, the desire to pursue research quickly can conflict with the time needed to build relationships and to engage communities. Additional strategies to accelerate and to modify the consent process for the unique conditions of a humanitarian emergency can overcome this conflict. These strategies are discussed below (S2 to S7).

There are important considerations when local community staff members are used in the research teams. Firstly, there are potential safety risks to community members who are later responsible to communicate to the community that the funding has stopped or that the trial has been stopped [52]. Secondly, the use of interpreters sometimes can increase the potential for incorrect communication of research objectives or potential risks [27]. This reinforces the importance of adequate training for research staff to be familiar with study objectives and risks.

### S2: Use alternative to standard written consent process

The traditional written consent process reinforces the power differential, and may exacerbate research participants’ security concerns [52]. Greater emphasis should be placed on information exchange between researchers and potential participants, with alternative ways to confirm consent [16, 29, 45, 55]. Research design should include procedures that enable potential participants sufficient time to consider participation and to discuss with family friends, and to be provided additional information about what the research involves after these discussions. These requirements may already exist with many research ethics boards [67], but one needs to be more deliberate to assure these procedures are followed, given researchers are often confronted with limited time in a humanitarian emergency. While this may delay initiation of a study whose researchers feel it should be rapidly initiated due to the humanitarian emergency, this process modification decreases the impact of participant vulnerability and socioeconomic status in the informed consent process [20, 63, 64, 68, 69]. 

Written consent process is inappropriate in low literacy areas [46, 51]. Use of audio and visual multimedia consent processes improve patient comprehension and information retention [70]. This includes multimedia videos, stories, pop-up definitions or quizzes [37, 51, 71]. Visual materials should include supplemental images of procedures and risks [46]. Household-based counselling in research participants’ homes may be more appropriate, and can promote cordiality [27]. Documentation of verbal informed consent with an illiterate person (or someone who doesn’t want to write consent) can be completed in the presence of a close relative [27]. Verbal consent may also be appropriate when a potential research participant has security concerns with written documentation of consent [72]. Verbal consent can sometimes be recorded, or in communities in which this not wanted, thumbprinting without written names can be considered [26]. Alternatively, researchers can document verbal consent by signing their own name to the consent document, and assign the research participant an interview identification number [72]. 

There are multiple versions of modified consent protocols from developed countries, which have not been assessed in humanitarian emergencies. These include third party consent, deferred consent, or waiving of consent documentation [73, 74]. The US Federal Policy for the Protection of Human Subjects permits the waiving of consent documentation under three circumstances. Firstly, a breach of confidentiality linking the research participant to the research could result in potential harm. Secondly, the research itself poses minimal risk. Thirdly, participants are members of a “distinct cultural group or community for whom signing documents is not the norm.” [75] Similar exceptions are noted in the International Ethical Guidelines for Health-Related Research Involving Humans by the Council for International Organizations of Medical Sciences (CIOMS) [76]. While the policies for consent documentation were not designed for humanitarian emergencies, each of the three conditions are commonly met in research completed in humanitarian emergencies, so it presents a potential option to overcome some of the challenges of written informed consent. However, waiving of consent should still require public consultations [77, 78] or evaluations of public discourse [79, 80]. 

Third party consent is acceptable in many studies in emergency medicine or critical care settings [81, 82], where patient acuity would make IRB approval delays disable research initiation. Deferred consent is considered more acceptable when the interventions are low risk [81], and of an emergency nature [83, 84]. However, neither third-party nor deferred consent have been evaluated in humanitarian emergencies, and thus remain difficult to recommend in such settings.

### S3: Modify traditional process of Research Ethics Board review

In Schopper e*t al*, the MSF IRB is described, which has three levels. For research that is of minimal risk (e.g. descriptive statistics), an expedited review process is used. For research of high risk, a less rapid “full IRB review” process is used, with the recognition that the review has to be prioritized given the emergent nature required for research deployment. There is also a “review exemption” for research around routine program implementation and assessment-related work. Interviews are sometimes not considered research by MSF, with people involved in interviews called “informants” rather than “research participants.” This IRB exemption is not granted if there is significant risk to the research participants. Interviewees are sometimes at risk of reexperiencing psychological trauma; in all such cases, IRB review is required. However, the MSF IRB recognizes that the application review process must be accelerated in research to be performed in humanitarian emergencies [45], as delays in IRB approval can be prohibitive [16, 58, 85]. IRBs elsewhere should similarly develop a more rapid review and approval process for research in humanitarian emergencies.

Researchers can complete pre-approved protocols or pre-formulated standard scripts for common surveys [86], which undergo local contextual change at the onset of a humanitarian emergency. This strategy has been successfully used in pandemics [87] and is also sometimes utilized by the MSF ethics committee [45]. Thus, inter-emergency activities focus on preparation of future research, by design and pre-approval of research protocols [82]. 

### S4: Dynamic consent

In traditional standard research trials, the informed consent procedure is completed once prior to the study, and then not formally revisited for the duration of the trial. However, in humanitarian emergencies, research participants’ risks change during the trial due to instabilities in social, economic, political, and health statuses. Therefore, the risks of ongoing participation in research trials also change. Researchers should therefore be required to check in with research participants to confirm the wish to continue in the research trial [29, 41]. On the other hand, many cross-sectional, survey or interview studies do not require a longitudinal relationship with research participants, thus negating the need for ongoing engagement to assess research risks.

Research studies may consider a “*situational adaptive design*” that builds adaptation into the study design in light of the potential changes in research participants’ risks throughout the humanitarian crisis [60]. This design may include planned check-ins, community meetings, question and answer sessions, or presentations, each of which provides an outlet for research participants and communities to express concerns during the trial [46]. The dynamic consent can be seen as an ongoing process^31^ that builds a “*partnership between researchers and participants*.” [2].

Eckenwiler et al. describes research studies in humanitarian settings taking on an adaptive “real-time responsiveness” quality, so that “real-time ethical attention” is required by researchers. This is defined as the capacity to be open to and to recognize research participants’ needs and concerns as they change throughout a humanitarian emergency. As these change, the risk to the study participants and community change, necessitating constant evaluation of the informed consent process [34]. 

### S5: Training of research staff in consent ethics

Homan identified four components for voluntary informed consent [88]. Firstly, all components of what will and might happen in the research study should be disclosed (information provision). Secondly, the participant should have the capacity to understand the information (capacity). Thirdly, the participants should be competent to make a rational judgment. Finally, a person’s agreement to participate should be voluntary, and free of undue influence or coercion. This is especially true in humanitarian emergencies, when potential research participants are more likely to be impacted by challenges that impair each of these conditions (Table 2). Recognizing whether these conditions are met needs to be done simultaneous to the informed consent process; thus, research staff should be trained in the ethics of informed consent.

Research staff who are soliciting informed consent in humanitarian emergencies should also be trained in active listening, which decreases power imbalance [41]. They should be able to identify people who struggle to make informed choices, such as children or people suffering from mental health conditions [16, 30, 55, 89]. They should learn to be explicit that research participation (or non-participation) has no impact on eligibility to receive aid or other services [46]. They should also assure that research participants can repeat back key concepts throughout the consenting process, including the voluntary nature of the process [46]. 

The United Nations Children’s Fund (UNICEF) Communities Care Program was designed to enhance understanding of gender-based violence in humanitarian situations of displacement and conflict. In an evaluation of the program, Glass et al. explained that local research staff had a 3 day training course to prepare to interview study participants. Local research staff were required to demonstrate understanding of research ethics principles, and to complete an online course. This preparation of research staff included review of informed consent, confidentiality, risks of study participation, building respect for study participants, and active listening. All research staff that conducted interviews with victims of gender-based violence (GBV) were trained in GBV prevalence, risk factors and services. This preparation for research staff training was extensive, and was built into an “inception phase”, prior to any data being collected [90]. 

### S6: Mandate transparency of commercial interests when present

Schopper et al. describes when a commercial tuberculosis diagnostic kit was compared to conventional culture methods. The risks to research participants were low, and the research investigators hadn’t intended on seeking informed consent to use patients’ sputum samples for the testing. Recognizing that the study was to enhance a technology for commercial potential, and that patients’ illness would eventually lead to corporate profits, the MSF IRB mandated a consent procedure that explicitly informed patients about the use of their sputum samples, and that the study was for commercial benefits [45]. It was noted that there was a community risk of dependence on a superior technology which may later become unaffordable when the study finished. Similarly, research participants should be fully informed if a profit entity is participating in the research study.

### S7: Mandate reporting of informed consent process in all publications

In a review of almost 500 research studies published in peer-reviewed journals regarding health in conflict, Ataullahjan et al. identified that obtaining ethics approval was reported in 48.2% studies, and obtaining informed consent in only 46.6% of studies [91]. A journal’s instructions to authors significantly improves reporting ethics approval and informed consent procedures [92]. While the lack of reporting doesn’t guarantee ethics approval or informed consent are not being completed, the potential for ethical violations in humanitarian disasters remains high [31]. “Mandating reporting of the informed consent process in all publications would provide reassurances that special considerations were made to overcome the challenges unique to humanitarian emergencies, such as the increased vulnerability of research subjects.” [91].

### Discussion: General

This comprehensive review identifies challenges in the informed consent process for research in humanitarian settings. While there is a paucity of evidence from humanitarian emergencies, this review has drawn from relevant contexts from emergency medicine, critical care medicine, and with vulnerable populations in developed nations. A template of challenges and strategies to overcome these challenges has been created, that can be used by researchers and IRBs to assure that research can be deployed in humanitarian settings safely, ethically, and with urgency. Low-resource settings may experience circumstances that do not qualify as a humanitarian emergency, but are similar enough that research faces the same challenges and potential solutions. Examples may include research on human trafficking, forced labor or forced marriage [93]. As such, the challenges and solutions identified in this comprehensive review may also be applicable to some situations outside of humanitarian emergencies.

This study has several strengths and weaknesses. Firstly, the study provides a comprehensive and practical guide regarding informed consent in research studies, that can be applied in a variety of humanitarian emergencies. However, the study did not differentiate which challenges or solutions were more applicable in specific emergencies. This could be evaluated in future research. Secondly, the study included 5 dabatases that included peer reviewed studies. While it is unlikely that this large number of databases searched excluded certain themes, it remains possible since grey literature was intentionally excluded.

This scoping review identifies challenges in the informed consent process in humanitarian emergencies, as well as evidence-based strategies to overcome these challenges. This information is particularly helpful to researchers, research ethics boards, and for non-governmental or governmental organizations that pursue research in humanitarian settings. This study can be used for creation of regulations and policies to complete research in humanitarian emergencies.

## Data Availability

Database is included in published paper, but raw data available on request to corresponding author.

## Abbreviations

CIOMS: Council for International Organizations of Medical Sciences
IRB: Institutional Research Board
LMIC: Low- and Middle-Income Countries
MSF: Médecins Sans Frontières, also known as Doctors Without Borders
UNICEF: United Nations Children’s Fund
